# Evaluation of Common Methods for Sampling Invertebrate Pollinator Assemblages: Net Sampling Out-Perform Pan Traps

**DOI:** 10.1371/journal.pone.0066665

**Published:** 2013-06-17

**Authors:** Tony J. Popic, Yvonne C. Davila, Glenda M. Wardle

**Affiliations:** 1 Desert Ecology Research Group, School of Biological Sciences, The University of Sydney, Sydney, New South Wales, Australia; 2 Long Term Ecological Research Network (LTERN), Sydney, New South Wales, Australia; Trinity College Dublin, Ireland

## Abstract

Methods for sampling ecological assemblages strive to be efficient, repeatable, and representative. Unknowingly, common methods may be limited in terms of revealing species function and so of less value for comparative studies. The global decline in pollination services has stimulated surveys of flower-visiting invertebrates, using pan traps and net sampling. We explore the relative merits of these two methods in terms of species discovery, quantifying abundance, function, and composition, and responses of species to changing floral resources. Using a spatially-nested design we sampled across a 5000 km^2^ area of arid grasslands, including 432 hours of net sampling and 1296 pan trap-days, between June 2010 and July 2011. Net sampling yielded 22% more species and 30% higher abundance than pan traps, and better reflected the spatio-temporal variation of floral resources. Species composition differed significantly between methods; from 436 total species, 25% were sampled by both methods, 50% only by nets, and the remaining 25% only by pans. Apart from being less comprehensive, if pan traps do not sample flower-visitors, the link to pollination is questionable. By contrast, net sampling functionally linked species to pollination through behavioural observations of flower-visitation interaction frequency. Netted specimens are also necessary for evidence of pollen transport. Benefits of net-based sampling outweighed minor differences in overall sampling effort. As pan traps and net sampling methods are not equivalent for sampling invertebrate-flower interactions, we recommend net sampling of invertebrate pollinator assemblages, especially if datasets are intended to document declines in pollination and guide measures to retain this important ecosystem service.

## Introduction

Different methods for sampling species composition fuelled the debate about the nature of communities [1], [2]. Ideological differences continue to influence the methods ecologists choose to sample assemblages and how they settle the eventual compromise between pragmatic issues and sound experimental design. The goal remains to design efficient and repeatable sampling methods that effectively represent the diversity of species and how their interactions vary over space and time. An emerging challenge is to ensure that datasets are also appropriate for subsequent comparative analyses such as comparing the structure of food webs based on mutualistic or antagonistic interactions [3]. Importantly, as species interactions are not fixed [4]–[6] records of presence will not establish interaction outcomes in a specific context.

Concern about the decline of pollination services has stimulated research and monitoring of pollinator assemblages and populations [7]–[9]. A range of sampling methods have been employed, but two, pan traps and net sampling, are considered effective at capturing the most species and highest abundance of pollinators [7], [8], [10], [11]. Pan traps are expected to capture greater species richness and abundance, be easier to use, lack collector bias and be cost-effective, whereas net sampling is often perceived to be more labor intensive, time consuming and subject to collector bias. However, the effectiveness of each method can depend on a range of factors including location of study due to vegetation type, resource (flowering) availability, and the composition of the pollinator community [12]–[14].

The emphasis on sampling as many species as possible may not necessarily be ideal or appropriate in all cases. Firstly, the relationship between pollinator populations and pollination is complex, and deficits in one do not necessarily equate to deficits in the other [15], [16]. An estimated 87.5% of flowering plant species are animal pollinated [17], and determining how pollinator population fluctuations affect pollination was recently identified as a key research question by pollination ecologists [18]. Secondly, invertebrates sampled using a pan trap or netted from a flower are not necessarily pollinators. For pollination to occur, a flower-visitor needs to pick up viable compatible pollen from a flower, travel to a conspecific plant, and deposit it on a receptive stigma [19]. Different methods supply different information about the pollination process and a species' functional role. Netting flower-visitors supplies information about the potential of that visitor as a pollinator, whereas without prior or subsequent investigation, pan traps provide limited information on species pollinating abilities. Depending on the study, having knowledge of species pollination function may be more useful in understanding the consequences of pollinator decline or the reasons for a reduction in pollination services.

It is timely that we evaluate how the different sampling methods affect the structure of the sampled assemblage. Community structure has direct consequences for ecosystem functioning [20], and more specifically, the network of interactions between plants and pollinators are structured in ways that increases the stability of ecological communities [3], [21], [22]. The structure of two closely linked assemblages, such as between pollinators and plants can also directly affect each another [23]. It is thus important to understand how different methods affect the realised pollinator assemblage, if we are to manage ecosystems to retain pollination function.

Pollinator population and assemblage data are valuable in determining functionally important species and guiding conservation and restoration programs. Monitoring is integral to research tracking the cause of population change, to identify the consequences of pollinator population declines, establish baselines, and form a core part to understanding the ecology and evolution of pollination. In a given study, the most appropriate sampling methods will depend on the aims, with consideration of available resources and time, and not necessarily which method is most effective at capturing most species. In this study, we aim to provide a quantitative comparison of the strengths and limitations of pan traps and net sampling.

We ask two main questions to frame our investigation: 1) What are the merits of each method, pan traps and net sampling, for discovery of species, understanding their pollination function within the system, tracking changes in abundance and composition over space and time and for contributing to global comparisons among habitats?, and 2) How does the spatial and temporal pattern of resource availability in terms of the number of plant species and level of flowering influence the effectiveness of each sampling method?

We approach these questions with a robust and extensive sampling regime for both pan traps and net sampling, and cover three sampling periods between June 2010 and July 2011 across a 5000 km^2^ area. We conducted our work in the Simpson Desert of Australia, an undisturbed ecosystem with no exotic invertebrate species, and an area rich in invertebrate diversity but lacking in-depth scientific investigation. We focus on all invertebrate species, and not only bees. Although bees are considered important pollinators globally [16], a key area for future pollination research is to more fully understand the roles played by the full complement of pollinators [17], [18]. We expect that net sampling and pan traps may differ in species discovery and abundance, but composition will differ as net sampling tracks floral resources more closely, making net sampling more functionally relevant to pollination.

## Materials and Methods

### Ethics statement

Plant and invertebrate collections were carried out under a Queensland government Scientific Purposes Permit (WISP07623410). Insect specimens are currently stored at The University of Sydney and the Australian Museum (AM) collection.

### Study system

The study was conducted in the north-eastern Simpson Desert, south-western Queensland, Australia, on Cravens Peak and Ethabuka private nature reserves. The dominant landforms are long parallel sand dunes, 8–10 m high, divided by interdune valleys (swales). The hummock grass, *Triodia basedowii* (Poaceae; spinifex) is dominant across the dune swales but less so on crests where shrub species (e.g. several *Acacia, Grevillea* and many in the Fabaceae) are common, along with a suite of ephemeral forbs and herbs that appear after rain. There are no introduced plant or flower-visitor species at the study sites, notably, no introduced European Honeybee *Apis mellifera* (Apidae). The Simpson Desert is classified as a hot, dry desert, with average annual rainfall between 100 mm and 150 mm. However, precipitation is highly variable by world standards [24]. Large, unpredictable rain events structure the environment and sustain high levels of biomass, creating relatively short ‘boom’ periods, immediately followed by relatively long ‘bust’ periods [25]. Detailed descriptions of the study area can be found in Popic, Wardle and Davila [5]. No permits were required for the described study, which complied with all relevant regulations. No protected species were sampled.

The study sites experienced several large rainfall events: 210 mm in March 2010, 160 mm in September 2010 and 470 mm in March 2011, which stimulated many plant species to flower. This, along with the vegetation structure (low and open) makes it a system suited to both pan trap and net sampling.

### Sampling design

A spatially-nested design, comprising three sites in the spinifex dominated dune fields [FR, KS, and MC, approximately 50 km apart, for details see 5], with two locations (1 km apart) at each site, was sampled. At each location, four 100 m transects, two on the dune crest and two in the swale, were placed to optimise representative flowering. Sampling occurred during the post-rain flowering period (14^th^–25^th^ June 2010, 12^th^–24^th^ November 2010, and 22^nd^ June –4^th^ July 2011) and included winter and summer flowering species.

### Invertebrate sampling

Net sampling – Flower-visiting invertebrates were sampled along each 100 m×5 m belt transect. Four collectors were deployed among the eight transects at each site: two at each location. Collectors sampled flower-visitors using nets from all plant species along transects for two hours and fulfilled these conditions: concurrent sampling of crest and swale between collectors, each transect sampled for 30 minutes by each collector, and collectors alternated dune zones. These measures helped negate any effect of collector bias. A visitation was defined as any physical contact between the animal and a flower. Flower-visitors were then caught using nets and plastic containers, and transferred into 5 mL vials for transportation. Plant species from which a visitor was collected was recorded.

Net sampling at each site occurred for three consecutive days in morning and afternoon sessions (June and July 1100–1300 and 1430–1630, November 900–1100 and 1500–1700), timing of which varied to best match the activity patterns of invertebrates, and to avoid extreme midday heat. To increase the representation at the transect and above levels, and to minimise any potential effects of weather on captures, sampling only occurred during fine weather, and pooled over the three day period. Sites were sampled in random order for each survey. A total of 432 hours was spent net sampling.

Pan traps – Pan traps were deployed along each 100 m transect during the three days of net sampling. Pans were made from polyethylene plastic bowls (400 mL, 110 mm diameter, 70 mm high) painted in either UV fluorescent yellow, blue or white paint (Educational Colours, Victoria, Australia). These colours have been demonstrated to be equally effective for capturing of a broad range of invertebrates in this habitat (Popic and Wardle unpublished data). Six pans (two of each colour) were placed along each transect, 15 m apart, in alternating colours. In each pan we placed 100 mL of detergent mixture (5 mL of non-odorous detergent in 1.5 L water). Pans were checked and cleared of captures at 1700 daily, and reset each morning. Therefore we removed any confounding effects of nocturnal captures in pan traps that would not be reflected in diurnal net sampling. Specimens were stored in 70% ethanol. In total, 1296 pan trap-days were deployed.

Invertebrates were viewed under a dissecting microscope and sorted to morphospecies. Bees were identified to species by taxonomist Michael Batley at the Australian Museum, Sydney.

### Plant diversity and flowering intensity

Vegetation along each transect was assessed during each sampling period (June 2010, November 2010, June 2011). Each plant species was scored between 0–5 (0-absent, 5-abundant) for flowering intensity and abundance of flowering plants. Species identifications were checked with reference sets for the study sites and vouchers specimens collected. Nomenclature follows Alexander [26] and Urban [27].

### Data analysis

Total species richness and abundance, and the richness and abundance of invertebrate orders [Hymenoptera - split into bees (Anthophila), ants (Formicidae), and wasps and sawflies (non-bee and non-ant Apocrita, and Symphyta, respectively, referred to as wasps for simplicity); Diptera; Lepidoptera] were compared using ANOVA with method (net, pan), sampling trip (June 2010, November 2010, July 2011), site (FR, KS, MC), locations (n = 2, nested within site), and dune zone (crest, swale) as factors. Captures of other orders were insufficient to compare statistically. Two ant species occasionally swarmed pan traps (in 1% of cases) so after examination of patterns, they were excluded from abundance comparisons to remove undue bias in the final results. Bias by either sampling method toward different bee families was investigated as previous research found differing ability in pollen transport by bee families in the study area [5].

Sampling effort was assessed using individual-based rarefaction curves. We estimated the cumulative number of species for each increase of 10 individuals in samples grouped by method, site, trip, location and dune zone using PRIMER-E v6 (*Plymouth Routines in Multivariate Ecological Research*).

Assemblage composition was investigated using a five-way nested PERMANOVA (maximum permutations  = 9999) with method, trip, site, location, and dune as factors. Terms with negative estimates of variation were removed by sequentially pooling terms and then re-evaluating the model [28]. Bray-Curtis similarity coefficients were used on square-root transformed data. When main effects were significant, pairwise tests were conducted to determine which levels of factors were responsible for significance. Monte Carlo sampling to determine *P*-values was used when the total number of possible permutations was low. To visually represent differences among assemblages, two-dimensional non-metric multidimensional scaling (nMDS) ordinations, based on Bray-Curtis dissimilarity coefficients, were produced using PRIMER-E v6. In order to investigate whether the core common species caught are similar between the two methods, the PERMANOVA and nMDS were repeated including only those species where more than 20 individuals were caught in total, thereby eliminating the contribution of rare species.

Linear regression analyses were used to compare for the two methods how flowering intensity, and flowering species richness, influenced the species richness and abundance of invertebrate samples. Data were log transformed to meet test assumptions.

To test whether the floral assemblage had a stronger relationship with the invertebrate assemblage sampled with nets or pan traps, we compared pairs of multivariate data (i.e. floral assemblage – pan trap invertebrate assemblage, floral assemblage – net sampled invertebrate assemblage) using the RELATE analysis in PRIMER-E. The test works by first determining the among-sample relationships (based on Bray-Curtis similarity coefficients) within each multivariate dataset (i.e. the two closest samples are determined, and then the next closest, and so forth). If the among-sample relationships agree in exactly the same way between pairs of multivariate datasets then the rank correlation coefficient (Spearman's ρ) will equal 1. If there is no relationship, then ρ will be approximately zero. The ρ value was compared by randomly permuting the sets of samples and recalculating ρ to create a frequency histogram [28].

## Results

### 1) What are the merits of each method for discovery of species, understanding their function within the system, tracking changes in abundance and composition over space and time and for contributing to global comparisons among habitats?

A total of 436 invertebrate species and 7294 specimens were collected using both methods (Table 1). Net sampling yielded 327 species and 4730 specimens that visited flowers of 61 plant species (Appendix S1), while pan traps collected 233 species and 2564 specimens. Of the 296 species with at least two captures in the combined total, 42.2% were caught only by nets, 15.9% only by pan traps, and 41.9% by both methods (of all 436 species, proportions were: 46.5%, 24.9%, and 28.6% respectively). Species caught with both methods accounted for 80.1% of the total abundance. From the total 7294 specimens, 140 singletons and 58 doubletons were sampled, while separately, 104 singletons and 42 doubletons from nets and 105 singletons and 32 doubletons from pan traps. Individual based rarefaction curves suggest sampling was sufficient for both methods (Appendix S2). Net sampling and pan traps performed similarly in terms of species discovery and abundance in July 2011, but nets caught more species and greater abundance in June 2010 and November 2010. Rarefaction curves suggest that a greater intensity of pan trap sampling in June 2010 and November 2010 would not increase species discovery to the level of net sampling (Appendix S2).

**Table 1:** Species richness and abundance of invertebrates sampled by net and pan sampling.

	Net		Pan		Total	
	Species	Abundance	Species	Abundance	Species	Abundance
Hymenoptera	213 (65)	3632 (77)	148 (64)	1749 (68)	278 (64)	5381 (74)
Bees	62 (19)	1758 (37)	42 (18)	1233 (48)	72 (17)	2991 (41)
Apidae	2 (<1)	77 (2)	3 (1)	111 (4)	3 (<1)	188 (3)
Colletidae	34 (10)	663 (14)	25 (11)	438 (17)	41 (9)	1101 (15)
Halictidae	13 (4)	962 (20)	10 (4)	678 (26)	15 (3)	1640 (23)
Megachilidae	13 (4)	56 (1)	4 (2)	6 (<1)	13 (3)	62 (<1)
Ants	18 (6)	320 (7)	21 (9)	141 (6)	26 (6)	461 (6)
Wasps	133 (41)	1534 (32)	85 (37)	375 (15)	180 (41)	1909 (26)
Coleoptera	26 (8)	150 (3)	18 (8)	103 (4)	38 (9)	253 (4)
Diptera	38 (12)	217 (5)	25 (11)	521 (20)	46 (11)	738 (10)
Hemiptera	34 (10)	200 (4)	18 (8)	59 (2)	45 (10)	259 (4)
Lepidoptera	10 (3)	421 (9)	12 (5)	103 (2)	15 (3)	524 (7)
Orthoptera	3 (<1)	3 (<1)	9 (4)	21 (<1)	10 (2)	24 (<1)
Thysanoptera	2 (<1)	103 (2)	2 (<1)	7 (<1)	2 (<1)	110 (2)
Other	1 (<1)	4 (<1)	1 (<1)	1 (<1)	2 (<1)	5 (<1)
Total	327	4730	233	2564	436	7294

Percentage shown in brackets.

Nets sampled a greater number of species of most orders and families than pan traps: about 50% more bees, wasps, Coleopteran and Dipteran species, and about twice as many Hemipteran species (Table 1), while pans sampled more ant, Lepidopteran and Orthopteran species. Pan traps sampled 58% of all bee species, while nets sampled 86%, including all Megachilidae, but missed one Apidae species, *Thyreus warooensis* which is parasitic on other bees (Appendix S3). The majority of species sampled by nets and pan traps were Hymenoptera (65.2% and 63.5% respectively), with wasps (40.7% and 36.5% respectively) the most speciose group, followed by bees (19.0% and 18.0% respectively, Table 1). Other orders were also similarly proportionally represented in nets and pan traps. Bees were the most abundant captures in nets and pan traps, comprising a relatively high proportion of pan trap captures (37.2% and 48.1% respectively). Wasps were abundant in net sampling but distinctly less so in pan traps (32.4% and 14.6% respectively). Nets also sampled higher abundance of Lepidoptera, and pans sampled many more Diptera.

**Figure 1:**
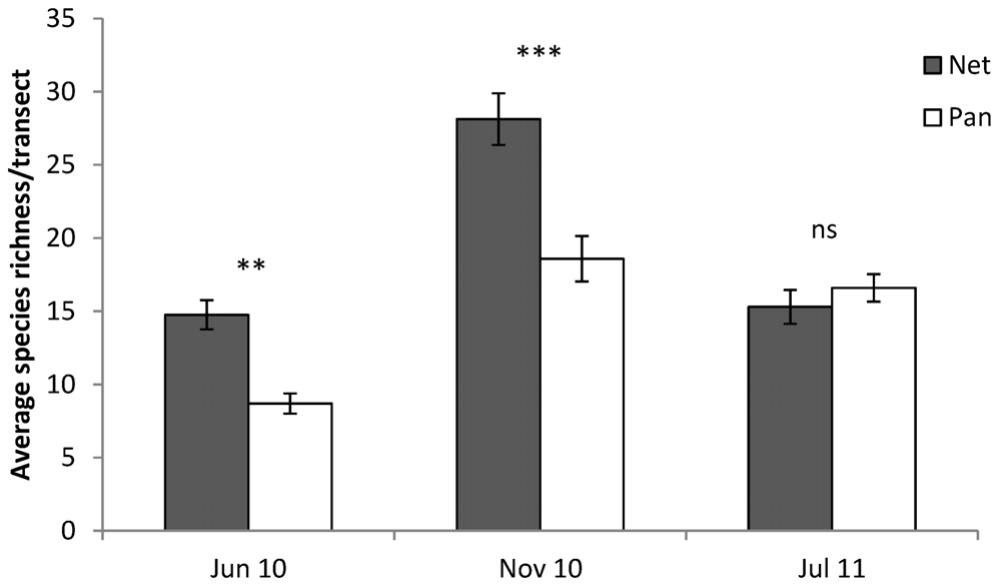
Species richness per transect of each method for the three sampling trips (average ±SE, n = 24). The method by trip interaction effect was significant (F_(2,6)_  = 33.35, P<0.01) as nets sampled more species in June and November 2010, but not in July 2011 where there was no difference. ***  = *P*<0.001, **  = *P*<0.01, ns  = *P*>0.05, significance determined with Tukey HSD test.

**Figure 2:**
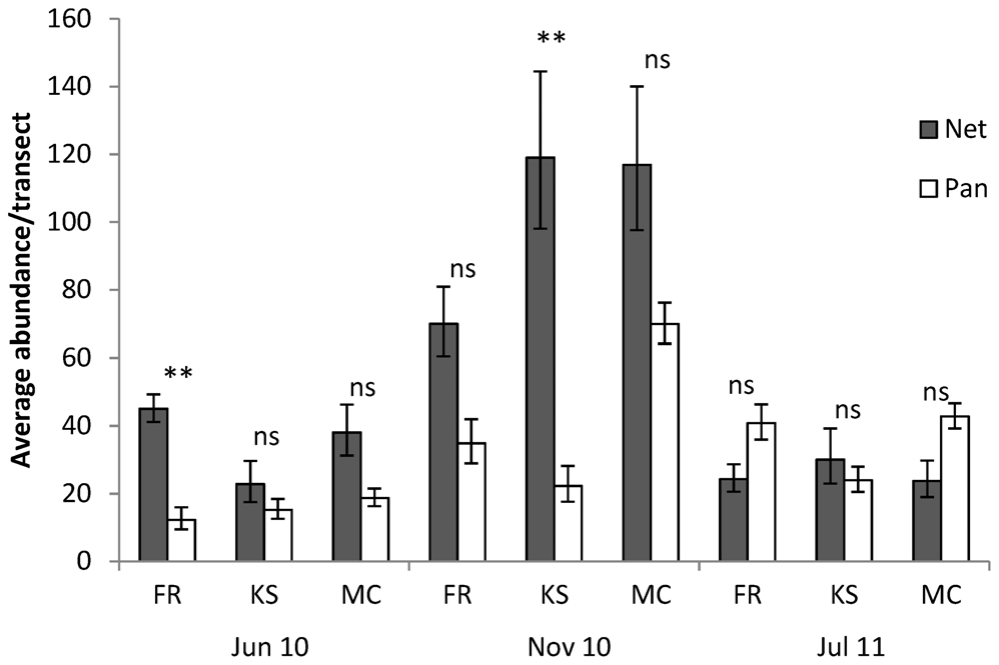
Invertebrate abundance per transect of each method at each site and trip (average ±SE, n = 8). An interaction effect between method, trip and site was found (F_(4,6)_  = 5.66, P<0.05). **  = *P*<0.01, *  = *P*<0.05, ns = *P*>0.05, significance determined with Tukey HSD test.

Species richness was influenced by the interaction between method and trip (F_(2,6)_  = 33.35, *P*<0.01); nets sampled significantly more species than pan traps in June 2010 (14.8±1.0 and 8.7±0.7 mean species ±SE per transect, respectively), and November 2010 (28.1±1.8 and 18.6±1.6 respectively) but methods were equivalent in July 2011 (15.3±1.2 and 16.6±0.9 respectively; Fig. 1, Appendix S4). Invertebrate abundance exhibited a significant three-way interaction between method, trip, and site (F_(4,6)_  = 5.66, *P*<0.05, S4): average abundance per transect was generally greater for net samples in June and November 2010 (nets sampled between 30% and 600% greater abundance), but in only two sites was the difference significant (Fig. 2). In July 2011, abundance from nets and pan traps were not statistically different (Fig. 2).

Bee species richness and abundance exhibited significant three-way interactions (method × site × trip, F_(4,6)_  = 18.11, *P*<0.01; F_(4,6)_  = 15.47, *P*<0.01 respectively), but there was no consistent response to method, among trip × site combinations (Appendix S5). Wasp species richness and abundance showed significant method × trip interactions (F_(2,6)_  = 10.65, *P*<0.05; F_(2,6)_  = 17.67, *P*<0.01 respectively): nets sampled greater species richness and abundance in June and November 2010, but methods were equal in July 2011 (Appendix S5). Dipteran abundance was greater in pan traps in November 2010 and July 2011 (Appendix S5) and the method × trip interactions was significant (F_(2,6)_  = 13.96, *P*<0.01). Method did not significantly affect Dipteran species richness (F_(2,6)_  = 3.85, *P*>0.05). Lepidopteran species richness and abundance were always significantly greater with net sampling (F_(1,3)_  = 30.92, *P*<0.05; F_(1,3)_  = 48.44, *P*<0.01 respectively; Appendix S5).

**Figure 3:**
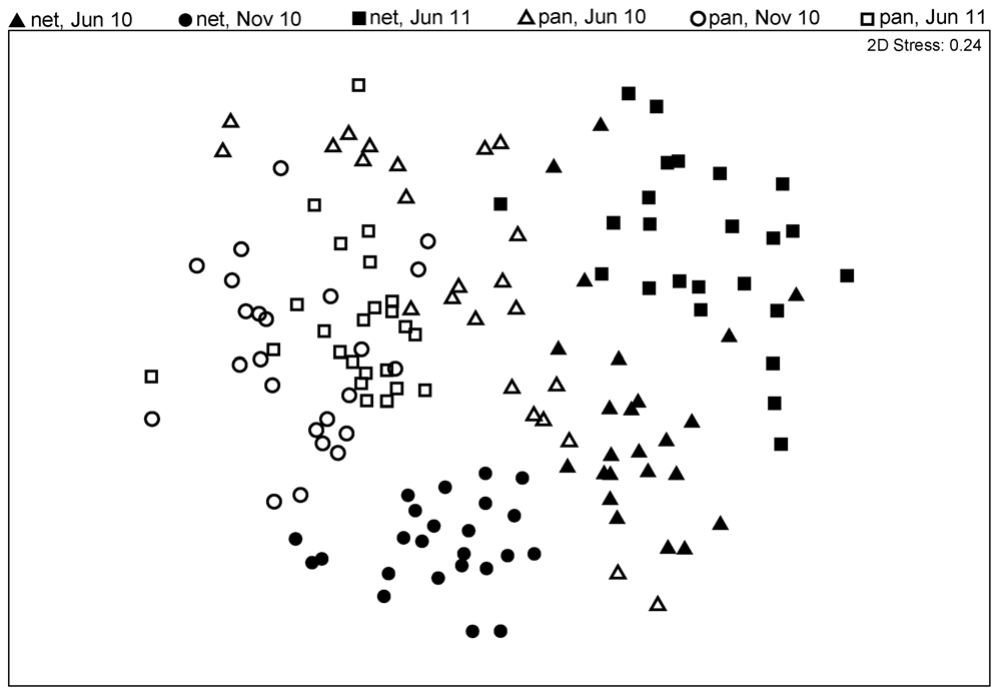
Nonmetric multidimensional scaling (nMDS) ordination of invertebrate assemblages sampled with nets and pan traps. Solid symbols represent nets, hollow symbols represent pans, and shapes represent the sampling periods.

The nMDS plot, grouped by method and trip for clarity indicates separation between assemblages caught by nets and pan traps (Fig. 3). The species composition differed significantly between net and pan trap samples (F_(1,105)_  = 22.885, *P*<0.001, Table 2; Fig. 3), among trips (F_(2,6)_  = 12.118, *P*<0.001, Table 2; Fig. 3), sites (F_(2,3)_  = 5.100, *P*<0.001, Table 2) and between locations (F_(3,105)_  = 1.282, *P*<0.05, Table 2). The three-way interaction term (method × trip × site) was also significant (F_(4,105)_  = 2.902, *P*<0.001, Table 2), and pairwise comparisons of all site by trip possibilities showed that the species composition of net and pan trap samples were significantly different (Appendix S6). All combinations of trips were significantly different from each other for both methods and all sites except for the net samples at KS and MC between June 2010 and July 2011 (Appendix S6). Sites differed for both methods and all trips except between all site combinations for net samples in July 2011, and for pan trap samples between FR and MC in July 2011 (Appendix S6). Similarly, the nMDS plot based on common species indicates separation of the assemblages caught by nets and pan traps (Appendix S7), which is also supported by a significant difference in species composition between net and pan trap samples using PERMANOVA (F_(1,81)_  = 24.90, *P*<0.001, Appendix S8).

**Table 2:** PERMANOVA results of main test assessing differences between invertebrate assemblages.

Source	df	MS	*Pseudo-F*	*P*(perm)
Method	1	51996	22.89	<0.001
Trip	2	31065	12.12	<0.001
Site	2	14857	5.10	<0.001^1^
Dune	1	2768	1.22	ns
Location(Site)	3	2913	1.28	<0.05
Method × Trip	2	17881	7.87	<0.001
Method × Site	2	8914	3.92	<0.001
Method × Dune	1	3378	1.49	<0.05
Trip × Site	4	9233	3.60	<0.001
Trip × Location(Site)	6	2564	1.13	ns
Method × Trip × Site	4	6591	2.90	<0.001
Method × Trip × Dune	2	2358	1.04	ns
Trip × Site × Dune	4	2460	1.08	ns
Method × Trip × Site × Dune	4	2610	1.15	ns
Residual	105	2272		

1 = *P*-value determined using Monte Carlo sampling.

### 2) How does the spatial and temporal pattern of resource availability in terms of the number of plant species and level of flowering influence the effectiveness of each sampling method?

**Figure 4:**
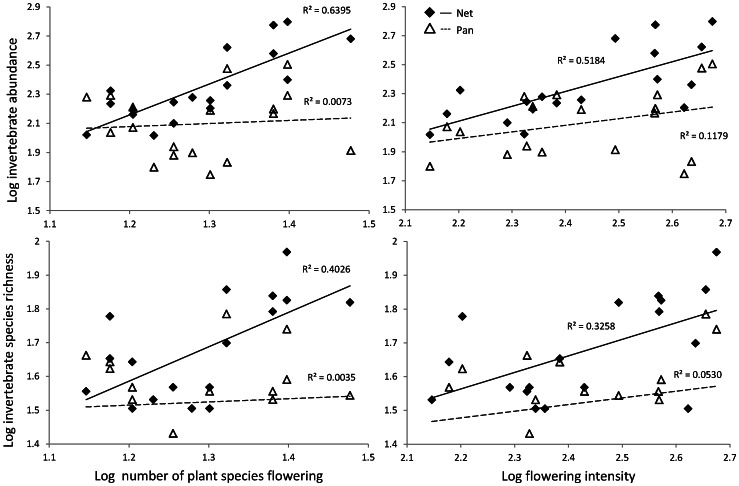
Relationship between invertebrate species richness and abundance, and flowering intensity and richness for both methods. Regression line fitted.

Flowering intensity and diversity positively increased the abundance and species richness of invertebrates sampled by nets (Fig. 4). The number of plant species flowering explained 64% of the variation in the invertebrate abundance (F_(1,16)_  = 28.39, *P*<0.001) and 40% of the variation in invertebrate species richness (F_(1,16)_  = 10.78, *P*<0.05). Flowering intensity explained less of the variation of invertebrate abundance and richness (r^2^  = 0.52, F_(1,16)_  = 17.22, *P*<0.01 and r^2^  = 0.33, F_(1,16)_  = 7.73, *P*<0.05 respectively; Fig. 4). By contrast, the species richness and abundance sampled by pans showed no significant relationship with flowering intensity or flowering species richness.

The invertebrate assemblage composition from net and pan traps showed some similarity with the floral composition (ρ = 0.28 for nets, p = 0.1% and, ρ = 0.11 for pans, p = 2.6%). The larger ρ indicates there was greater similarity in the relationship among samples of the net sampled dataset and floral assemblage dataset.

## Discussion

Our goal was to provide guidance on the reasons to choose between two common methods, pan traps and net sampling, for sampling pollinator populations and assemblages. Our evidence suggests that these two methods are not equivalent. We therefore evaluate their relative merits below, in terms of the quantitative measures used in our comparison, and also in qualitative terms as summarised in Table 3.

**Table 3:** Summary of pros and cons for invertebrate pollinator pan trap and net sampling techniques based on this study.

	Pan traps	Net sampling
Study purpose:		
Monitoring invertebrate populations of familiar study area	✓✓	✓✓
To determine species function	X	✓✓^1^
Pollination function:		
Plant visitation	X	✓✓
Visitor behaviour	X	✓✓
nteraction frequency	X	✓✓
Allows pollen analysis	X	✓✓
Method logistics:		
Sampling during little flowering	✓✓	X
Optimise preparation time	✓	✓
Minimal collection time	✓✓	X
Condition of collection material	X	✓✓
Ease of Invertebrate sorting	X	✓✓
Cost	✓	✓
No collector experience	✓	X^2^

✓✓ - Ideal;

✓- suitable;

X - not suitable

1 – net sampling usually required and ideal but not sufficient to determine visitor function.

2 – This can be effectively managed to make it suitable.

Net sampling was superior to pan trap sampling at species discovery and quantifying abundance, mostly during periods of increased flowering. In addition, the rarefaction curves indicate that more pan trapping would not increase species discovery to the level of net sampling. Our findings contrast other studies in which pan traps have been found to capture a greater species richness and abundance of bees [7], [8], [29]. Although we included all invertebrates in our comparison, nets sampled greater species richness and abundance of most taxonomic groups. Bee species richness and abundance did not show a consistent response to sampling method, and pans sampled a greater abundance of Diptera, which potentially include important pollinators [16]. Wasps were frequent flower-visitors but under-represented in pan traps, and our observational records suggest many are likely pollinators. Megachilidae bees were also poorly represented in pan traps, and these constitute a functionally important group, efficient at pollen-transport [5]. The local flowering distribution may contribute to the superiority of net sampling, highlighting that local flowering patterns are important to consider when sampling pollinators. Flowering was usually focused along dune crests, and so sampling along transects was effective at capturing the full floral diversity and abundance, enabling efficient collection of flower-visitors with nets. Conversely, pan traps passively sample invertebrates, and are not restricted to flower-visitors like net sampling. Pan traps are also not restricted to flower-visitors of plants species on a transect, but can sample an area dependent on the flight range of invertebrate species, which is usually less than several hundred meters for solitary bees [30].

In addition to the differences in species richness and abundance, each method also differed in species composition. Compositional difference between methods does not support the assumption that the invertebrate assemblage caught with pan traps is equivalent to the pollinator assemblage, as net sampling captures flower-visiting invertebrates, species likely to be functionally important for the plant species from which they were sampled [5], even if they are not pollinators [19]. Previous studies report high similarity in species composition between assemblages caught by pan and net sampling [8], [29], while others report different assemblage composition [11], [14]. Pan traps may not catch the same species as netting due to behavioral avoidance of pans by certain taxa (e.g. Megachilidae, Lepidoptera) coupled with pans attracting species that were not recorded visiting flowers (e.g. some ants and flies). Of particular note is that species sampled in pan traps were under-representative of generalist flower-visitor species compared to the net-sampled species. Therefore, pans are not sampling the functionally important species that contribute to the stability of flower-visitor interaction networks and potentially pollination [21], [31].

Invertebrates sampled with nets more closely tracked floral resources than invertebrates from pan traps, further supporting that net-sampled invertebrates are functionally important in pollination. The effectiveness of pan traps showed no response to floral resources which contrasts findings of other studies [11], [12], [32]. An increase in floral resource diversity is expected to increase the invertebrate diversity as the specific dietary requirements of more species will be met, and indeed a positive relationship is frequently observed in flower-visitor networks [33], [34]. The lack of response from pan traps is thus surprising, and does not support the notion that the invertebrate assemblage caught with pan traps is the pollinator assemblage. However, as pan trap capture rates are independent of floral resources, they are ideal to use during low, and potentially across varying, resource levels. To track long-term changes in pollinators, it is important to consider the numerous relatively short-term natural fluctuations in pollinator assemblages and populations, to which resource variation contributes substantially [16], [35], [36]. Net sampling would require the effects of local resource-driven variation in pollinator populations to be taken into account, to allow comparisons across spatial and temporal scales. However, floral resources should be considered in pollination studies regardless of the sampling method, as the flowering plant assemblages and populations will largely drive the geographic units of functional relevance of pollinator diversity [18].

Pan traps and net sampling also differ in a variety of qualitative aspects (Table 3), in addition to the quantitative responses discussed above, such as how each method contributes to understanding species function. Unlike pan traps, net sampling allows easy collection of additional data necessary to determine species function, for example visitation rate, visitor behavior, resource handling, and analysis of visitor pollen loads [5], [37]. With these data, we can determine the two main components of pollinator effectiveness: frequency and effectiveness of interactions [19]. Pan traps offer no information on visitation or pollen-transport, but despite this, pan traps are potentially useful in areas where the pollination biology and pollination function of species are already well studied and known. Inferring function needs to be performed cautiously, however, as interactions are not functionally fixed in time and space [6].

We found the total effort involved with each method comparable (Table 3), contrasting previous studies [8], [11]. Preparation for net sampling involved sourcing field equipment (nets, vials, and killing jars), whereas for pan traps, involved sourcing materials and the application of paint onto pans, which was a considerable effort as many pans were required. The open, low vegetation at our study sites allowed pan traps to be set on the ground, but in most vegetation types, it is necessary for pans to be suspended above ground level [8], adding to preparation time. In the field, our study required the daily removal of captures from pan traps, which is itself no quick task, and because of the dry, hot conditions, pan traps required daily refilling, amounting to 60 minutes each day. Net sampling required the allotted catching time, which requires more time in the field than setting up pan traps, and specimens also needed to be transferred into transportation vials at the end of each day. The poor condition of pan trap specimens was also a negative feature, and required additional processing. Droege [38] has outlined techniques to improve the condition of wet (pan trap) specimens including the use of preservatives in pan traps, but we found specimens that were originally netted (dry) in far better condition, making them easier and quicker to process and identify, and more desirable for catalogue. Collector experience is an important component of the suitability of net sampling, particularly for catching small and/or fast-moving insects, but we found it was effectively managed with short training, and appropriate study design, for example, rotating collectors to minimise any bias, and collecting all flower-visitors, annulling decision making by the collector.

### Conclusions on choosing the best method

Previous investigations provide useful insights and guidelines to determine and design the most appropriate method to sample pollinators [7], [8], [10], [29]. When choosing a sampling method, we must be sure pollinator species are being sampled. Information about that species' pollination function (or their ecological role in the community generally), is therefore important, and will be valuable when guiding conservation and restoration programs. However, knowing which species are pollinators is difficult to determine without detailed investigations, and functional differences between the two methods are therefore important. We base our evaluation of pan trap and net sampling not only on which method samples the most species or greatest abundance of bees, but the entire pollinator assemblage, and how effectively the sampled assemblages can be related to pollination function.

We found net sampling to be superior at capturing greatest species richness and abundance, but this depended on floral resource levels. Other studies have found pan traps more effective at collecting the species richness than net sampling, but often only bees were considered and differences may lie in the intensity of net sampling [8], [29]. Net sampling was only slightly more time-consuming after preparation and specimen processing (particularly of wet pan traps) was considered. Collector experience and bias will need to be managed, but this can be done effectively to minimise effects. The key and important difference between the two methods is in relation to pollination function aspects of the assemblages that each method samples. We found the methods sampled different assemblages even when the two methods captured similar species richness and abundance during low floral resources, and the information supplied by nets to be an important and necessary component in determining species function. These are important differences for two reasons: we need to be sure we are sampling the relevant species in an area, and the relationship between pollinator populations and pollination services is complex and reductions in one does not necessarily result in reductions in the other. Given these differences in functional interpretation, we recommend net sampling as the preferred way to sample invertebrate pollinator populations and assemblages.

## Supporting Information

Appendix S1 **Abundance of invertebrates caught visiting flowers of each plant species using net sampling at each site and during each sampling period.** Plant names follow the Australian Plant Name Index.
(DOCX)

Appendix S2 **Rarefaction curves for pan trap (broken line) and net sampling (solid line).** Transects within each dune zone at each location at each site were pooled and used to generate average estimates (n = 12, ±SE).
(TIF)

Appendix S3 **List of bee species and abundance sampled by pans and nets.**
(DOCX)

Appendix S4 **Partially nested ANOVA results comparing species richness and total abundance between methods (log transformed).**
(DOCX)

Appendix S5 **Species richness and abundance per transect (n = 24) of invertebrate orders (average ±SE).** Hymenoptera split in bees, ants and wasps. Bees are grouped by Method x Trip x Site so mean values are from n = 8 transects.
(TIF)

Appendix S6 **Pairwise comparisons for the significant ANOSIM method (a) x trip (b) x site (c) interaction comparing invertebrate assemblages collected using net sampling and pan traps.**
(DOCX)

Appendix S7 **Nonmetric multidimensional scaling (nMDS) ordination of assemblages of common invertebrate species.** Common invertebrates were arbitrarily defined as any species with a total abundance of 20 or greater of which there were 61 invertebrate species. Solid symbols represent nets, hollow symbols represent pans, and shapes represent the sampling periods.
(TIF)

Appendix S8 **Comparison of assemblages of common invertebrate species sampled using PERMANOVA and illustrated with nMDS plot.** Common invertebrates were arbitrarily defined as any species with a total abundance of 20 or greater of which there were 61 invertebrate species.
(DOCX)
