# Supplementary material for: Evaluation of Common Methods for Sampling Invertebrate Pollinator Assemblages: Net Sampling Out-Perform Pan Traps
Source: PLoS One. 2013 Jun 17;8(6):e66665. doi: 10.1371/journal.pone.0066665 (PMC3684574; doi:10.1371/journal.pone.0066665)
Supplement: Appendix S1 — Abundance of invertebrates caught visiting flowers of each plant species using net sampling at each site and during each sampling period. Plant names follow the Australian Plant Name Index. (DOCX) [file pone.0066665.s001.docx]

**Appendix S1.** Abundance of invertebrates caught visiting flowers of each plant species using net sampling at each site and during each sampling period. Plant names follow the Australian Plant Name Index.

|  |  | **Jun-10** | | |  | **Nov-10** | | |  | **Jul-11** | | | **Total abundance** | **Total species richness** |
| --- | --- | --- | --- | --- | --- | --- | --- | --- | --- | --- | --- | --- | --- | --- |
| **Family** | **Plant species** | **FR** | **KS** | **MC** |  | **FR** | **KS** | **MC** |  | **FR** | **KS** | **MC** |  |  |
| Aizoaceae | *Trianthema pilosa* | 26 |  | 59 |  | 3 | 1 | 63 |  |  |  |  | 152 | 35 |
| Amaranthaceae | *Alternanthera* sp. A |  |  |  |  | 2 |  |  |  |  |  |  | 2 | 1 |
| Amaranthaceae | *Ptilotus atriplicifolius* |  |  | 3 |  |  |  |  |  |  |  | 12 | 15 | 5 |
| Amaranthaceae | *Ptilotus latifolius* | 1 |  |  |  | 15 | 2 | 34 |  |  | 15 | 2 | 69 | 27 |
| Amaranthaceae | *Ptilotus polystachyus* | 7 |  | 11 |  | 18 |  | 12 |  |  |  |  | 48 | 11 |
| Araliaceae | *Trachymene glaucifolia* | 103 | 91 | 120 |  | 13 | 19 | 47 |  |  |  |  | 393 | 70 |
| Asteraceae | Asteraceae sp. A |  |  |  |  |  |  | 1 |  |  |  |  | 1 | 1 |
| Asteraceae | *Calotis erinacea* |  | 9 |  |  | 1 | 129 |  |  | 1 | 37 | 6 | 183 | 35 |
| Asteraceae | *Calotis plumothera* |  |  | 2 |  |  |  |  |  |  |  |  | 2 | 2 |
| Asteraceae | *Helichrysum* sp. A |  |  | 2 |  |  |  |  |  |  |  | 1 | 3 | 3 |
| Asteraceae | *Helipterum molle* |  |  |  |  | 6 | 25 |  |  |  |  |  | 31 | 6 |
| Asteraceae | *Myriocephalus* sp. A |  |  |  |  |  |  | 1 |  |  |  |  | 1 | 1 |
| Asteraceae | *Pterocaulon sphacelatum* |  |  |  |  |  |  | 31 |  |  |  | 52 | 83 | 21 |
| Boraginaceae | *Halgania cyanea* | 7 |  |  |  |  |  |  |  |  |  |  | 7 | 6 |
| Boraginaceae | *Trichodesma zeylanicum* | 19 | 1 | 11 |  | 7 |  | 27 |  | 55 | 21 | 18 | 159 | 28 |
| Brunoniaceae | *Brunonia australis* |  |  |  |  | 6 | 13 |  |  |  |  |  | 19 | 12 |
| Chenopodiaceae | Chenopodiaceae sp A |  |  |  |  |  |  |  |  |  |  | 6 | 6 | 1 |
| Chenopodiaceae | *Salsola kali* | 1 |  |  |  |  |  |  |  |  |  |  | 1 | 1 |
| Euphorbiaceae | *Adriana tormentosa* |  |  |  |  | 8 |  |  |  |  |  |  | 8 | 2 |
| Fabaceae | *Acacia bivenosa* |  | 19 |  |  |  | 22 |  |  |  | 24 |  | 65 | 17 |
| Fabaceae | *Acacia coriacea* |  | 3 |  |  |  |  |  |  |  |  |  | 3 | 3 |
| Fabaceae | *Acacia dictyophleba* | 50 | 39 |  |  |  |  |  |  | 19 | 34 | 32 | 174 | 25 |
| Fabaceae | *Acacia ligulata* | 60 | 2 |  |  |  |  |  |  | 138 | 27 | 93 | 320 | 43 |
| Fabaceae | *Acacia stenophylla* |  |  |  |  |  |  |  |  | 1 |  |  | 1 | 1 |
| Fabaceae | *Crotalaria cunninghamii* |  |  |  |  | 2 |  | 4 |  | 34 | 38 | 84 | 162 | 23 |
| Fabaceae | *Crotalaria eremaea* |  | 1 | 10 |  | 8 | 10 | 84 |  | 22 | 28 | 30 | 193 | 61 |
| Fabaceae | *Crotalaria smithiana* |  |  |  |  |  |  | 4 |  |  |  | 12 | 16 | 1 |
| Fabaceae | Fabaceae sp. A |  |  | 3 |  |  |  |  |  |  |  |  | 3 | 2 |
| Fabaceae | *Psoralea eriantha* |  |  |  |  |  | 2 |  |  |  |  |  | 2 | 2 |
| Fabaceae | *Senna artemisioides* |  |  |  |  |  | 2 |  |  |  |  |  | 2 | 1 |
| Fabaceae | *Senna pleurocarpa* |  |  |  |  |  | 9 |  |  |  | 2 |  | 11 | 8 |
| Fabaceae | *Swainsona phacoides* | 1 |  |  |  |  |  |  |  |  |  | 2 | 3 | 2 |
| Fabaceae | *Tephrosia rosea* |  | 5 | 4 |  | 65 | 44 | 42 |  | 1 | 9 |  | 170 | 30 |
| Frankeniaceae | *Frankenia gracilis* |  |  |  |  |  |  | 7 |  |  |  |  | 7 | 4 |
| Goodeniaceae | *Goodenia cycloptera* | 24 | 14 | 12 |  | 28 | 29 | 26 |  | 17 | 14 | 7 | 171 | 30 |
| Goodeniaceae | *Lechenaultia divaricata* |  |  |  |  |  |  | 10 |  |  |  |  | 10 | 4 |
| Goodeniceae | *Scaevola depauperata* | 3 | 1 | 21 |  | 82 | 8 | 47 |  | 1 |  |  | 163 | 29 |
| Goodeniceae | *Scaevola parvibarbata* |  | 4 |  |  |  | 25 | 3 |  |  |  |  | 32 | 10 |
| Goodeniceae | *Scaevola parviflora* | 19 |  |  |  |  | 5 |  |  |  | 1 |  | 25 | 11 |
| Haloragaceae | *Haloragis gossei* | 4 |  |  |  |  |  |  |  |  |  |  | 4 | 1 |
| Lamiaceae | *Dicrastylis costelloi* |  |  |  |  | 92 | 6 |  |  | 15 |  |  | 113 | 41 |
| Lamiaceae | *Dicrastylis llewellynii* |  |  |  |  |  |  |  |  |  | 11 |  | 11 | 8 |
| Lamiaceae | *Newcastelia cephalantha* |  |  |  |  |  |  |  |  | 16 | 122 |  | 138 | 26 |
| Lamiaceae | *Newcastelia spodiotricha* |  |  |  |  | 146 | 172 |  |  |  |  |  | 318 | 36 |
| Malvaceae | *Abutilon otocarpum* |  |  |  |  |  | 43 |  |  |  |  |  | 43 | 1 |
| Malvaceae | *Rulingia loxophylla* |  |  |  |  | 1 |  |  |  |  |  |  | 1 | 1 |
| Malvaceae | *Sida fibulifera* |  | 2 |  |  | 16 | 18 | 29 |  |  |  |  | 65 | 26 |
| Malvaceae | *Sida trichopoda* |  |  |  |  | 5 | 2 |  |  |  |  |  | 7 | 6 |
| Malvaceae | *Triumfetta winneckeana* |  |  |  |  | 3 | 68 |  |  |  |  |  | 71 | 10 |
| Myrtaceae | *Eucalyptus pachyphylla* |  |  |  |  |  | 316 |  |  |  |  |  | 316 | 18 |
| Myrtaceae | *Euphorbia drummondii* | 74 | 57 | 132 |  | 70 | 88 | 220 |  | 130 | 35 | 69 | 875 | 132 |
| Portulacaceae | *Calandrinia balonensis* |  |  | 11 |  | 26 | 14 | 81 |  |  |  |  | 132 | 23 |
| Proteaceae | *Grevillea juncifolia* | 8 |  |  |  | 1 |  |  |  |  |  | 6 | 15 | 6 |
| Proteaceae | *Grevillea stenobotrya* |  |  |  |  |  |  | 221 |  |  | 61 | 3 | 285 | 37 |
| Rubiaceae | *Oldenlandia pterospora* |  |  |  |  |  | 8 |  |  |  |  |  | 8 | 6 |
| Sapindaceae | *Atalaya hemiglauca* |  |  |  |  |  |  |  |  | 1 |  |  | 1 | 1 |
| Scrophulariaceae | *Eremophila macdonnellii* |  |  |  |  | 12 |  | 68 |  |  |  | 1 | 81 | 13 |
| Scrophulariaceae | *Eremophila* sp. A |  |  |  |  |  |  | 2 |  |  |  |  | 2 | 2 |
| Solanaceae | *Solanum* sp A |  |  | 10 |  |  |  | 3 |  |  |  |  | 13 | 4 |
| Violaceae | *Hybanthus aurantiacus* |  |  |  |  |  | 2 |  |  |  |  |  | 2 | 2 |
|  | Unknown 1 |  |  |  |  |  | 3 |  |  |  |  |  | 3 | 2 |
|  | Total | 407 | 248 | 411 |  | 636 | 1085 | 1067 |  | 451 | 479 | 436 | 5220 | 327 |

The five shades of cell colour indicate flowering intensity (darker shade equates to greater flowering intensity) of that species at that specific time and place.
