# Supplementary figures and images for: Evaluation of Common Methods for Sampling Invertebrate Pollinator Assemblages: Net Sampling Out-Perform Pan Traps
Source: PLoS One. 2013 Jun 17;8(6):e66665. doi: 10.1371/journal.pone.0066665 (PMC3684574; doi:10.1371/journal.pone.0066665)

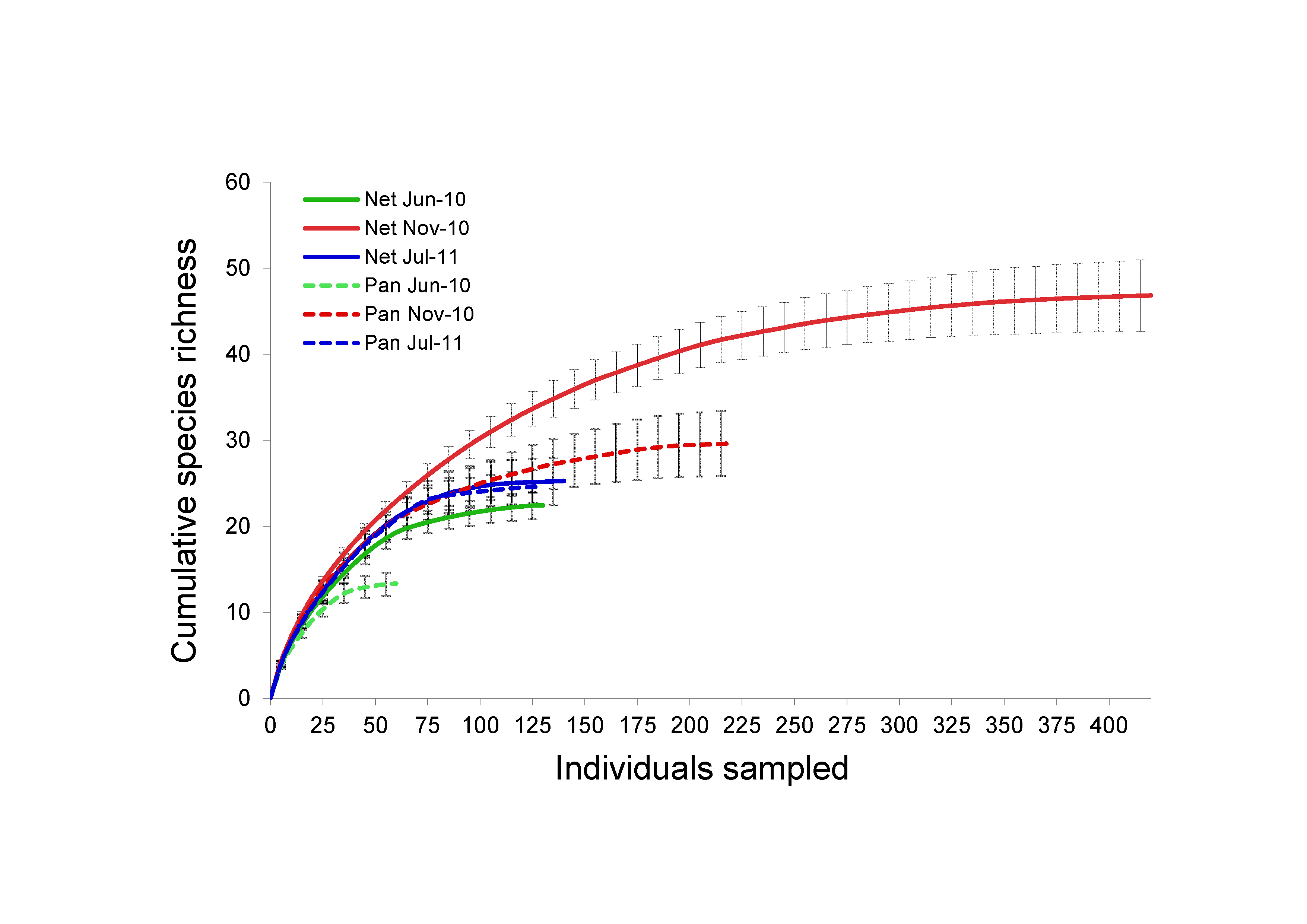

Supplement: Appendix S2 — Rarefaction curves for pan trap (broken line) and net sampling (solid line). Transects within each dune zone at each location at each site were pooled and used to generate average estimates (n = 12, ±SE). (TIF) [file pone.0066665.s002.tif]

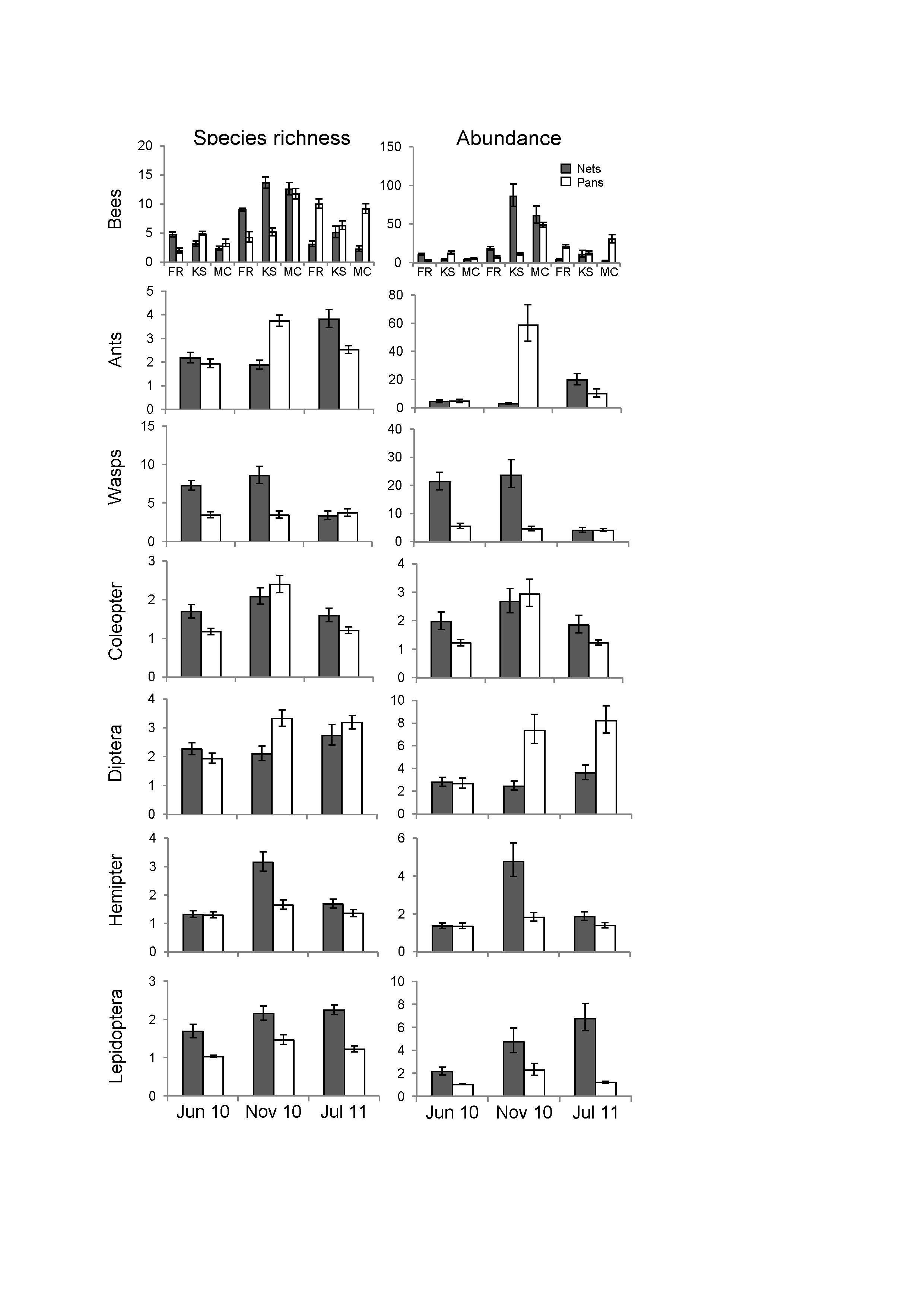

Supplement: Appendix S5 — Species richness and abundance per transect (n = 24) of invertebrate orders (average ±SE). Hymenoptera split in bees, ants and wasps. Bees are grouped by Method x Trip x Site so mean values are from n = 8 transects. (TIF) [file pone.0066665.s005.tif]

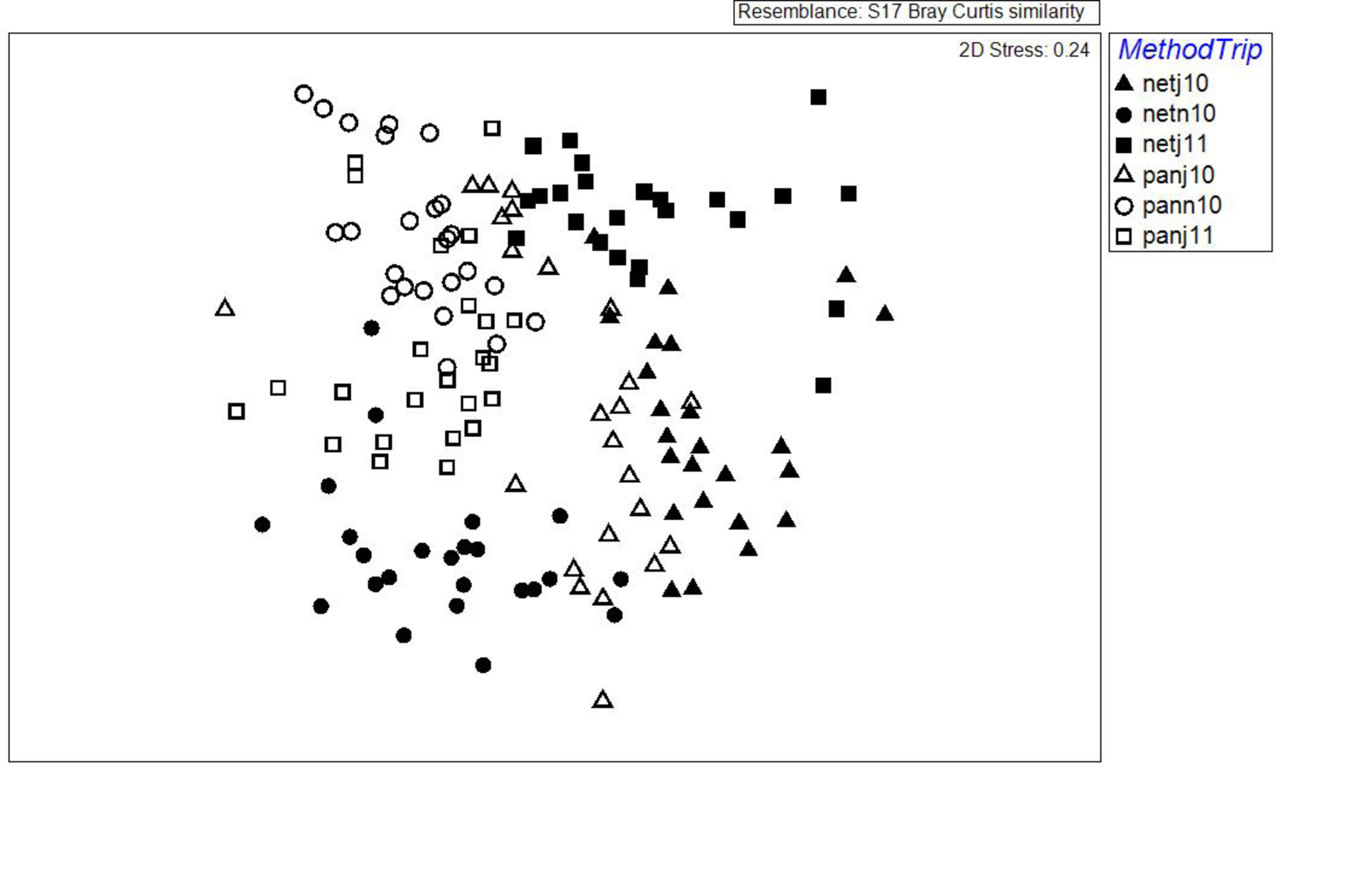

Supplement: Appendix S7 — Nonmetric multidimensional scaling (nMDS) ordination of assemblages of common invertebrate species. Common invertebrates were arbitrarily defined as any species with a total abundance of 20 or greater of which there were 61 invertebrate species. Solid symbols represent nets, hollow symbols represent pans, and shapes represent the sampling periods. (TIF) [file pone.0066665.s007.tif]
