# Supplementary material for: Evaluation of Common Methods for Sampling Invertebrate Pollinator Assemblages: Net Sampling Out-Perform Pan Traps
Source: PLoS One. 2013 Jun 17;8(6):e66665. doi: 10.1371/journal.pone.0066665 (PMC3684574; doi:10.1371/journal.pone.0066665)
Supplement: Appendix S3 — List of bee species and abundance sampled by pans and nets. (DOCX) [file pone.0066665.s003.docx]

**Appendix S3.** List of bee species and abundance sampled by pan traps and nets.

| **Family** | **Species** | **Net sampling** | **Pan traps** |
| --- | --- | --- | --- |
| Apidae | *Amegilla chlorocyanea* | 19 | 55 |
| Apidae | *Exoneurella eremophila* | 58 | 53 |
| Apidae | *Thyreus warooensis* |  | 3 |
| Colletidae | *Brachyhesma barrowensis* | 44 |  |
| Colletidae | *Chrysocolletes strangomeles* | 2 |  |
| Colletidae | *Euhesma loorea* |  | 1 |
| Colletidae | *Euhesma* sp. D | 31 | 14 |
| Colletidae | *Euhesma* sp. J |  | 9 |
| Colletidae | *Euhesma* sp. T | 2 | 3 |
| Colletidae | *Euhesma sybilae* | 18 | 1 |
| Colletidae | *Hylaeus albimandibulatus* | 2 |  |
| Colletidae | *Hylaeus albozebratus* | 65 | 13 |
| Colletidae | *Hylaeus calandriniae* | 12 | 33 |
| Colletidae | *Hylaeus circinatus* | 3 |  |
| Colletidae | *Hylaeus* sp. AB |  | 1 |
| Colletidae | *Hylaeus* sp. AG |  | 1 |
| Colletidae | *Hylaeus* sp. AH |  | 3 |
| Colletidae | *Hylaeus* sp. E | 3 | 1 |
| Colletidae | *Hylaeu*s sp. F | 75 | 1 |
| Colletidae | *Hylaeus* sp. H | 3 | 11 |
| Colletidae | *Hylaeus* sp. K | 9 |  |
| Colletidae | *Hylaeus* sp. L |  | 1 |
| Colletidae | *Hylaeus* sp. Z | 12 | 3 |
| Colletidae | *Hylaeus trimerops* | 13 |  |
| Colletidae | *Hylaeus vittatifrons* | 4 |  |
| Colletidae | *Hylaeus wyndhamensis* | 26 |  |
| Colletidae | *Hylaeus zebrinus* | 4 | 48 |
| Colletidae | *Leioproctus alloeopus* | 7 |  |
| Colletidae | *Leioproctus argentifrons* | 2 | 1 |
| Colletidae | *Leioproctus finkei* | 13 | 39 |
| Colletidae | *Leioproctus impatellatus* | 8 | 2 |
| Colletidae | *Leioproctus lucidicinctus* | 63 | 8 |
| Colletidae | *Leioproctus sequax* | 98 | 174 |
| Colletidae | *Leioproctus* sp. A | 25 | 61 |
| Colletidae | *Leioproctus* sp. AD |  | 2 |
| Colletidae | *Leioproctus* sp. AE | 29 | 3 |
| Colletidae | *Leioproctus* sp. B | 4 |  |
| Colletidae | *Leioproctus* sp. S | 17 |  |
| Colletidae | *Leioproctus* sp. V | 1 |  |
| Colletidae | *Leioproctus* sp. W | 4 | 4 |
| Colletidae | *Leioproctus tarsalis* | 3 |  |
| Colletidae | *Xanthesma flava* | 16 |  |
| Colletidae | *Xanthesma lucida* | 42 |  |
| Colletidae | *Xanthesma nigrior* | 3 |  |
| Halictidae | *Homalictus sphecodopsis* | 33 |  |
| Halictidae | *Homalictus urbanus* | 340 | 346 |
| Halictidae | *Lasioglossum alacarinatum* | 105 | 97 |
| Halictidae | *Lasioglossum albopilosum* | 2 | 24 |
| Halictidae | *Lasioglossum ebeneum* | 5 |  |
| Halictidae | *Lasioglossum immaculatum* | 217 | 41 |
| Halictidae | *Lasioglossum platychilum* | 120 | 82 |
| Halictidae | *Lassioglossum cognatum* |  | 27 |
| Halictidae | *Lassioglossum eremaean* | 18 | 40 |
| Halictidae | *Lipotriches australica* | 41 | 3 |
| Halictidae | *Lipotriches flavoviridis* | 73 | 15 |
| Halictidae | *Lipotriches semipallida* A | 4 |  |
| Halictidae | *Lipotriches* sp 89 | 3 |  |
| Halictidae | *Lipotriches* sp AC |  | 3 |
| Halictidae | *Lipotriches* sp G | 1 |  |
| Megachilidae | *Coelioxys reginae* | 5 | 1 |
| Megachilidae | *Megachile apicata* | 2 |  |
| Megachilidae | *Megachile aurifrons* | 1 |  |
| Megachilidae | *Megachile barvonensis* | 3 |  |
| Megachilidae | *Megachile bidentis* | 2 |  |
| Megachilidae | *Megachile boharti* | 2 |  |
| Megachilidae | *Megachile captionis* | 2 | 3 |
| Megachilidae | *Megachile giddioorla* | 11 |  |
| Megachilidae | *Megachile maculariformis* | 3 |  |
| Megachilidae | *Megachile sequior* | 17 | 1 |
| Megachilidae | *Megachile serricauda* | 6 | 1 |
| Megachilidae | *Megachile tarltoni* | 1 |  |
| Megachilidae | *Megachile warrambuccis* | 1 |  |
