# Supplementary material for: Evaluation of Common Methods for Sampling Invertebrate Pollinator Assemblages: Net Sampling Out-Perform Pan Traps
Source: PLoS One. 2013 Jun 17;8(6):e66665. doi: 10.1371/journal.pone.0066665 (PMC3684574; doi:10.1371/journal.pone.0066665)
Supplement: Appendix S4 — Partially nested ANOVA results comparing species richness and total abundance between methods (log transformed). (DOCX) [file pone.0066665.s004.docx]

**Appendix S4.** Partially nested ANOVA results comparing species richness and total abundance between methods (log transformed).

|  |  | Species richness | | | Total abundance | | |
| --- | --- | --- | --- | --- | --- | --- | --- |
| Source | df | MS | *F* | *P* | MS | *F* | *P* |
| Method | 1 | 817.0 | 27.32 | <0.05 | 1.634 | 38.00 | <0.01 |
| Trip | 2 | 1662.9 | 53.64 | <0.001 | 2.375 | 91.35 | <0.001 |
| Site | 2 | 117.3 | 4.60 | ns | 0.314 | 9.81 | <0.05 |
| Dune | 1 | 1094.5 | 243.22 | <0.01 | 0.381 | 11.55 | <0.05 |
| Location(site) | 3 | 25.5 | 1.42 | ns | 0.032 | 0.74 | ns |
| Method x Trip | 2 | 366.8 | 33.35 | <0.01 | 1.058 | 27.84 | <0.01 |
| Method x Site | 2 | 44.6 | 1.49 | ns | 0.175 | 4.07 | ns |
| Method x Dune | 1 | 5.1 | 0.11 | ns | 0.165 | 0.75 | ns |
| Method x Location(site) | 3 | 29.9 | 1.67 | ns | 0.043 | 1.00 | ns |
| Trip x Site | 4 | 179.8 | 5.80 | <0.05 | 0.079 | 3.04 | ns |
| Trip x Dune | 2 | 154. | 8.24 | <0.05 | 0.121 | 6.72 | <0.05 |
| Trip x Location(site) | 6 | 31.0 | 1.73 | ns | 0.026 | 0.60 | <0.05 |
| Site x Dune | 2 | 66.1 | 14.69 | <0.05 | 0.124 | 3.76 | ns |
| Dune x Location(site) | 3 | 4.5 | 0.25 | ns | 0.033 | 0.77 | ns |
| Method x Trip x Site | 4 | 22.4 | 2.04 | ns | 0.215 | 5.66 | <0.05 |
| Method x Trip x Dune | 2 | 42.3 | 1.51 | ns | 0.081 | 0.90 | ns |
| Method x Trip x Location(Site) | 6 | 11.0 | 0.61 | ns | 0.038 | 0.88 | ns |
| Method x Site x Dune | 2 | 42.3 | 0.95 | ns | 0.022 | 0.10 | ns |
| Method x Dune x Location(site) | 3 | 44.5 | 2.49 | ns | 0.220 | 5.12 | <0.01 |
| Trip x Site x Dune | 4 | 30.9 | 1.65 | ns | 0.033 | 1.83 | ns |
| Trip x Dune x Location(Site) | 6 | 18.7 | 1.04 | ns | 0.018 | 0.42 | ns |
| Method x Trip x Site x Dune | 4 | 3.2 | 0.11 | ns | 0.028 | 0.31 | ns |
| Method x Trip x Dune x Location(Site) | 6 | 28.1 | 1.57 | ns | 0.090 | 2.09 | ns |
| Error | 72 | 17.9 |  |  | 0.043 |  |  |
