# Supplementary material for: Evaluation of Common Methods for Sampling Invertebrate Pollinator Assemblages: Net Sampling Out-Perform Pan Traps
Source: PLoS One. 2013 Jun 17;8(6):e66665. doi: 10.1371/journal.pone.0066665 (PMC3684574; doi:10.1371/journal.pone.0066665)
Supplement: Appendix S6 — Pairwise comparisons for the significant ANOSIM method (a) x trip (b) x site (c) interaction comparing invertebrate assemblages collected using net sampling and pan traps. (DOCX) [file pone.0066665.s006.docx]

**Appendix S6**. Pairwise comparisons for the significant ANOSIM method (a) x trip (b) x site (c) interaction comparing invertebrate assemblages collected using net sampling and pan traps.

| **Pairwise Test** | **t** | ***P*** |  | **t** | ***P*** |  | **t** | ***P*** |
| --- | --- | --- | --- | --- | --- | --- | --- | --- |
| **Method x Trip x Site** |  |  |  |  |  |  |  |  |
| 1. **Net, Pan** | **FR** | |  | **KS** | |  | **MC** | |
| Jun 10 | 3.344 | <0.01 |  | 3.140 | <0.01 |  | 2.483 | <0.05 |
| Nov 10 | 3.366 | <0.01 |  | 2.913 | <0.01 |  | 2.142 | <0.05 |
| Jul 11 | 2.979 | <0.05 |  | 2.800 | <0.01 |  | 2.160 | <0.05 |
| 1. **Net** |  |  |  |  |  |  |  |  |
| Jun 10, Nov 10 | 2.392 | <0.05 |  | 2.777 | <0.01 |  | 2.365 | <0.05 |
| Jun 10, Jul 11 | 2.594 | <0.05 |  | 1.476 | ns |  | 1.997 | ns |
| Nov 10, Jul 11 | 2.758 | <0.05 |  | 2.777 | <0.05 |  | 2.055 | <0.05 |
| **Pan** |  |  |  |  |  |  |  |  |
| Jun 10, Nov 10 | 2.192 | <0.05 |  | 2.738 | <0.01 |  | 3.961 | <0.01 |
| Jun 10, Jul 11 | 2.627 | <0.05 |  | 2.597 | <0.05 |  | 2.862 | <0.05 |
| Nov 10, Jul 11 | 2.649 | <0.05 |  | 1.978 | <0.05 |  | 2.382 | <0.05 |
|  |  |  |  |  |  |  |  |  |
| 1. **Net** | **Jun 10** | |  | **Nov 10** | |  | **Jul 11** | |
| FR, KS | 1.969 | <0.05 |  | 2.403 | <0.01 |  | 1.593 | ns |
| FR, MC | 2.023 | <0.05 |  | 1.800 | <0.05 |  | 1.310 | ns |
| KS, MC | 2.071 | <0.05 |  | 1.779 | <0.05 |  | 1.165 | ns |
| **Pan** |  |  |  |  |  |  |  |  |
| FR, KS | 3.525 | *<*0.001 |  | 1.746 | <0.05 |  | 1.784 | <0.05 |
| FR, MC | 3.117 | <0.01 |  | 2.628 | <0.01 |  | 1.577 | ns |
| KS, MC | 3.212 | <0.01 |  | 1.961 | <0.05 |  | 1.875 | <0.05 |
