# Supplementary material for: Evaluation of Common Methods for Sampling Invertebrate Pollinator Assemblages: Net Sampling Out-Perform Pan Traps
Source: PLoS One. 2013 Jun 17;8(6):e66665. doi: 10.1371/journal.pone.0066665 (PMC3684574; doi:10.1371/journal.pone.0066665)
Supplement: Appendix S8 — Comparison of assemblages of common invertebrate species sampled using PERMANOVA and illustrated with nMDS plot. Common invertebrates were arbitrarily defined as any species with a total abundance of 20 or greater of which there were 61 invertebrate species. (DOCX) [file pone.0066665.s008.docx]

**Appendix S8.** Comparison of assemblages of common invertebrate species sampled using PERMANOVA. Common invertebrates were arbitrarily defined as any species with a total abundance of 20 or greater of which there were 61 invertebrate species.

| Source | df | MS | *Pseudo-F* | *P*(perm) |
| --- | --- | --- | --- | --- |
| Method | 1 | 40968 | 24.90 | <0.001 |
| Trip | 2 | 29644 | 12.16 | <0.001 |
| Site | 2 | 14785 | 5.06 | <0.001^1^ |
| Dune | 1 | 5127 | 2.29 | ns |
| Location(Site) | 3 | 2919 | 1.77 | <0.01 |
| Method x Trip | 2 | 23330 | 14.18 | <0.001 |
| Method x Site | 2 | 11886 | 7.23 | <0.001 |
| Method x Dune | 1 | 5444 | 2.46 | <0.05 |
| Trip x Dune | 2 | 3314 | 1.60 | ns |
| Trip x Site | 4 | 9534 | 3.91 | <0.001 |
| Site x Dune | 2 | 3540 | 1.58 | ns |
| Trip x Location(Site) | 6 | 2439 | 1.48 | <0.01 |
| Dune x Location(Site) | 3 | 2238 | 1.36 | ns |
| Method x Site x Dune | 2 | 4228 | 1.91 | ns |
| Method x Trip x Site | 4 | 7351 | 4.47 | <0.001 |
| Method x Trip x Dune | 2 | 2851 | 1.47 | ns |
| Method x Dune x Loc(site) | 3 | 2213 | 1.35 | ns |
| Trip x Site x Dune | 4 | 2508 | 1.21 | ns |
| Method x Dune x Loc(site) | 6 | 2067 | 1.26 | ns |
| Method x Trip x Site x Dune | 4 | 2948 | 1.52 | ns |
| Method x Trip x Dune x Lo(site) | 6 | 1933 | 1.18 | ns |
| Residual | 81 | 1645 |  |  |

^1^ = *P­*-value determined using Monte Carlo sampling.
